# A comparative study of serological diagnosis of Dengue outbreak 2019

**DOI:** 10.4314/ahs.v21i3.20

**Published:** 2021-09

**Authors:** Muhammad Bilal Habib, Noreen Sher Akbar, Anber Saleem

**Affiliations:** 1 National Institute of Health Islamabad Pakistan 44000; 2 DBS&H CEME National University of Sciences and Technology, Islamabad, Pakistan; 3 School of Dentistry, Shaheed zulfiqar ali Bhutto medical university Islamabad

**Keywords:** Dengue, NS-1, IgG, IgM, Immunochrometographic test

## Abstract

**Purpose:**

The interpretation & correlation of the different laboratory parameters in positive dengue cases in order to evaluate that which laboratory test is more significant for diagnosis of Dengue.

**Methods:**

Prospective examination of samples (patients' serum) for dengue virus of different genotype by using multiplex anti-dengue IgM, IgG. We have done NS-1 test by (ICT) immunochromatographic devices, and complete blood picture (CBC) by Sysmex XP-100.

**Result:**

Detection of Viral RNA in 100 patients showed effects in the total of 73 (73.0%) samples. This graphical comparison shows the whole positive cases including dengue NS-I antigen, dengue serology (IgM & IgG), total 62 positive cases of NS-I are detected, 10 positive cases of dengue IgM and 9 positive cases of IgG detected, in which Complete Blood Test (CBC) shows remarkable reduction in Platelets (32 cases) and Leucopenia in (24 positive cases).

**Conclusion:**

In this research, it is concluded that the diagnosis of dengue cases is preliminary limited to initial stages i.e. CBC or sometimes dengue NS-I, as dengue IgM severity is more effective than that of Dengue NS-I & IgG. Many patients who had negative results in CBC and NS-1 testing, became positive when IgM and IgG serology testing has been done.

## Background

Mosquito born disease commonly known as Dengue mainly caused by viruses which are serologically interrelated but antigenically diverse single stranded positive RNA virus. Genus *Flavivirus* (Family Flaviviridae) has four serotypes which are DENV-I, DENV-II, DENVIII & DENV-IV. Dengue virus lives in the clean environment therefore people living in clean environment are mostly prone to infections. Dengue Virus is most common in geographically distribution of Asian Countries 1. There is foremost part of ecological factors like temperature for the survival of adult vector. In effective periods these strains require optimum temperature. Indication of the situation is the most infectious disease in world's major health problem list distressing tropical & subtropical areas. This Arbovirus in most common in world's most populated areas in terms of morbidity & mortality. According to W.H.O approximations, more than 2.5 billion people are at the risk of dengue infection 2. In recent studies from District Swat of KPK, Pakistan 6376 cases were confirmed as Dengue and 23 deaths occurred in that area. Dengue Virus is lethal for all over the world's population and now adays this is huge problem for Public Health Correspondence. The occurrence of dengue virus is mainly observed in endemic areas especially in tropical and sub-tropical regions. According to a research, approximately 50 million people infected annually in more than 100 countries. In worldwide distribution of this infection the observed regions include Asia, America, Middle East & Africa. The severity of infection depends upon the environmental factors of that region 3.

In case of overall population, the Dengue Virus comes repeatedly per annum with more effectiveness once again as reported by W.H.O 4. It is widely observed in American states that there are DENV-2 & DENV-3 genotypes which were found more commonly and have known to be less virulent than genotypes of the Asia having same serotype. As showed by compact growth in both mosquitoes and culture Wang proved that DENV-III domain may play a part in viral adaptation to undeveloped hosts, the epidemiological studies reveals that the genotypes with higher virulent effect are lashing out the virus strains in lesser epidemiological impact 5. In current years, substantial consideration has focused mainly towards characterizing the scope of genetic diversity in the four dengue virus serotypes, predominantly through phylogenetic analyses and sequencing of envelope (E) gene already sequenced from strains that were sampled globally 6. Four serological types of dengue virus are DEN I, II, III and IV. It causes different primary and secondary infections^7^.

There are three main markers for the detection of Dengue Virus i.e. Dengue NS-1 ICT/ ELISA, Dengue Serology (IgM & IgG). In an acute infection with the early symptoms, IgM levels may increase which may occur within 3-5 days and persists 30 – 60 days after the infection exposure. IgG levels may also be raised in chronic infection type after 10 to 14 days of the infection and it will be detectable throughout life span. The technique which is used, called Hem agglutination Inhibition Assay 8. There are two main vectors responsible for the dengue infections first are *Aedes aegypti* and second are *Aedes albopictus*. In this form, *A. aegypti* is less active vector, although it is responsible for the infections in endemic regions. In advanced diagnosis, PCR plays an important role in specific detection of dengue (RNA) virus 9. Most of researchers concluded that very small part of infected patients may have immunoglobins M (IgM) in first 3 to 4 days after onset of symptoms. The nature of outbreaks may be advanced or worst for immune-compromised persons depending upon the effectiveness of the serotypes & strains of dengue virus^10^. The main root cause of the dengue virus is *Aedes aegypti*. The morbidity & mortality rates and economic costs are affected by the increasing incidence of dengue infection each year and still there is a raised number found from all over the world 11.

The main root cause of this fatal infection is DENV. Genus *Flavivirus*, family: Flaviviridae. The Dengue Virus is currently known as Mosquito-borne Flavivirus because of its recurrent infections such as yellow fever, dengue fever, dengue hemorrhagic fever & dengue shock syndrome which recorded in last decade. The specific dengue virus can be detected directly in sera by using PCR techniques or by cell culture supernatants detection in infected mosquito larvae 12. As it is observed that dengue is broad spectrum disease sometimes it reveals unrecognized signs & symptoms which may be misdiagnosed co-relating with the clinical presentations of the patient i.e. fever causes may mislead to another causative agents. There is variation in incubation period as in some patients there is sudden onset of symptoms and patient may suffer many early complications i.e. myalgia, anorexia, sore throat, headache and often macular skin rash 13. Most people experience self-limiting clinical course which may not lead them to severity of the infection like DHF & DSS. Unfortunately, there is no any presence of dengue vaccine or any specific antiviral therapy all over the world. The need of the hour is to discover the antidote. Due to the recent deprived disease surveillance, there is low reporting level, low case casualty rate, complications in diagnosis, and impulsive comparative analyses, impact of dengue and true incidence is expressively higher than which is currently reported apart 14. It is widely observed that dengue virus also infects the people like international travelers, physicians and doctors in western countries are most provoked with travel acquired dengue infections. Recently this infection is declared as the most important Arboviral illness in human beings. More than 100 tropic and sub-tropic countries are endemic in infection^15^. This study is aimed on diagnostic evaluation, to interpret & correlate the clinico-hematological parameters in positive dengue cases.

## Materials and Methods

Total number of 100 blood samples is collected from patients. Following are the materials and methods which are used in diagnosis of dengue fever on the basis of clinical signs & symptoms;

1. Complete Blood Count
2. Dengue NS-1 Antigen
3. Dengue Serology (IgM, IgG)

### Complete Blood Count

CBC is performed on fully automated hematology analyzer SYSMEX XP-100 series. It is state of the art machine from Japan which gives 21 parameters.

### Requirements

Sysmex XP-100, Microscope, Microscope Slides, Oil Immersion, Stains, Applicator Stick, Rotator.

### Procedure

Collect the specimen by vein-cut procedure in characterized anticoagulant vacutainer. Rotate the sample on rotator for 10 minutes. Suction the sample by the test and let it investigate. Spot a perfect 3 x 1-inch glass slide on a level surface (stationary slide), one with a frosted end. Blend the blood well. With a fine spreader, move a drop of blood around 2 to 3 mm in distance across to the stationary slide about ¼-inch from the end of the frosted on a similar side as the composing hand. Hold the end of the stationary slide inverse the blood drop with the non-composing hand. Position a spreader (non-frosted) slide at a 25 to 30-degree point to the stationary slide and bring it once more into the drop of blood. Permit the blood to spread along the rear of the spreader slide. Label the slide at the frosted end with the Patient recognizable proof number and date, utilizing a pencil or a precious stone pen. Air-dry the blood quickly however completely (a few minutes) before recoloring. You can do this physically or with a cool air blower. Stain the slide utilizing document recoloring. Leave for 10 minutes and wash the smear with faucet water and permit the smear to air dry. Perform smear examination and report the outcomes appropriately.

### Dengue NS-1 Antigen

The Dengue NS-1 Antigen test is a single step immunochromatographic test (ICT) for the rapid diagnosis of Dengue infection NS1 antigen in human serum, plasma or Whole blood. This test is advised as a guide in diagnosis of early dengue infection.

### Requirements

Patient's Serum, ICT Device, Buffer, Dropper/Pipette, Stopwatch

### Procedure

Bring all test devices, reagents and samples to room temperature for 15 minutes prior to performing the assay. Use a fresh test device for every sample. The device is not reusable. Just prior to use, remove the required number of Dengue NS1 test devices from their wrapper and place them on a flat surface area. Label the test unit with the patient name or identification number. Take 100 µl (3 drops) of Whole blood or serum/plasma with the included disposable pipette or a micropipette and add into the sample well. Interpret test results at 15∼20 minutes. Do not interpret the test results after 20 minutes, this can give false results.

### Dengue IgM

Dengue IgM is advised for in-vitro qualitative detection of Dengue IgM Antibodies in human serum or plasma and is used as a screening test for testing of collected blood samples suspected for Dengue. The kit detects all four subtypes; DEN1, DEN2, DEN3 & DEN4 of Dengue Virus.

### Dengue IgG

Dengue IgG is designed for in-vitro qualitative detection of Dengue IgM Antibodies in human serum or plasma and is used as a screening test for testing of collected blood samples suspected for Dengue.

## Results

### Clinical Findings

**Figure 1:**
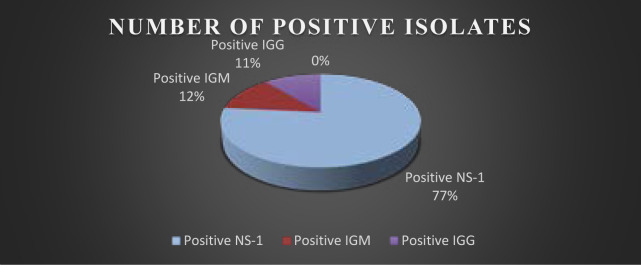
Total Number of Positive Isolates

Total 100 samples were investigated through diagnostics test (Dengue NS-1, Dengue Serology IgM & IgG) which shows 77% positive patients with NS-I, 12 % positive cases with Dengue IgM and 11 % Dengue IgG cases were detected as showed in Figure 1.

**Figure 2:**
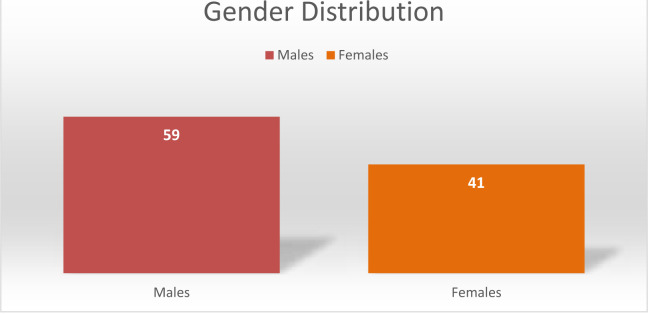
Gender/Sex Wise Distribution

Figure 2 showed the gender wise distribution of total number of samples. In which the ration of dengue positive cases in (59 %) and total ration of positive (cases is 41%).

**Figure 3:**
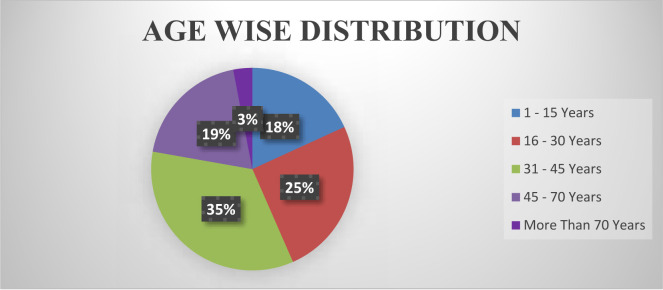
Age Wise Distribution

According to the graphical distribution of all ages showed in Figure 3. the ration in 1–15 years is (18 %), the ratio in 16–30 years is (25%), the ratio in 31–45 years is (35%), the ratio in 45–70 years is (19%) and the ratio in more than 70 years is (3%).

### Diagnostic Findings

**Figure 4:**
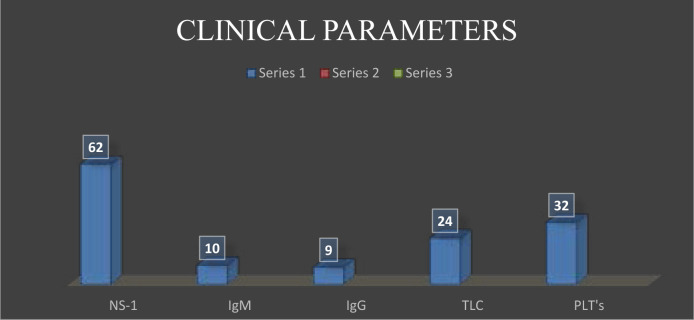
Clinical Comparison of Lab Parameters

This graphical comparison showed in Figure 4. the whole positive cases including dengue NS-I antigen, dengue serology (IgM & IgG), total 62 positive cases of NS-I are detected, 10 positive cases of dengue IgM and 9 positive cases of IgG are detected. In which Complete Blood Test (CBC) shows remarkable reduction in Platelets (32 cases) and Leucopenia in 24 positive cases which is used as diagnostic tool for the confirmation of dengue presence.

We analysed the comparison of NS-1 with Dengue IgM, the blue line upper than the cut of line shows the positive cases of NS-1 and orange line upper than the cutoff line shows the positive cases of IgM. We observed that Sample number 48, 49, 54, 55, 60, and 65 had negative results for NS-1, but there Dengue serology IgM results were positive as showed in Figure 5a, and 5b.

**Figure 5a:**
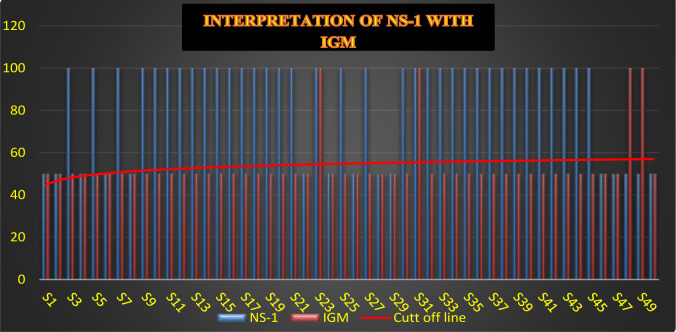
Clinical Interpretation Dengue NS-1 and Dengue IgM of Total Samples (1–50)

**Figure 5b:**
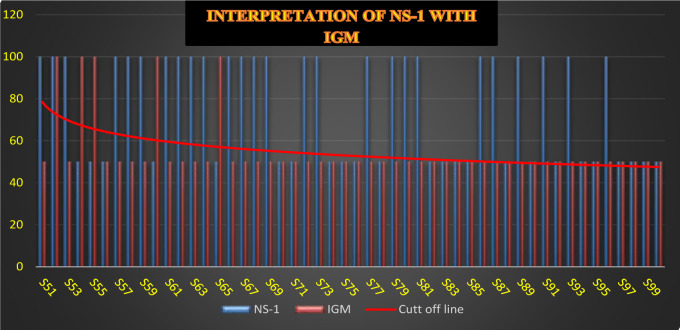
Clinical Interpretation Dengue NS-1 and Dengue IgM of Total Samples (51–100)

## Discussion

In this study, the male population was greatly affected (59 %) than female (41 %) while the infection rate was less in children; same study was reported by researchers in which the prevalence in males is (72%) which are more effective than females and children 1. The highest concentrations were reported in the America in which male population is highly effected. According to this study, from all the serotypes of dengue virus DENV-I & DENV-II serotypes are most frequently isolated. Same findings of isolated serotypes were DENV-1 and DENV-2 (90s) and DENV-2 and DENV-3 (2000–2007)^16^. The result of this study showed that in majority of cases blood CP were normal and NS-1 were negative but IgM and IgG of these patients became positive, another study result showed IgM levels elevated in acute infections and persists in blood for 2 months, and in chronic cases IgG level also raised after 14 days and detectable throughout life 1. Noteworthy geographic extension has been combined with quick increments in episode cases, pestilences, and hyperendemicity, prompting the more serious types of dengue. Transmission of dengue is currently present in each World Health Organization (W.H.O) locale of the world and in excess of 125 nations are known to be dengue endemic. The genuine effect of dengue all-inclusive is hard to find out because of variables, for example, deficient sickness reconnaissance, misdiagnosis, and low degrees of detailing. Presently accessible information likely terribly thinks little of the social, monetary, and illness trouble. Assessments of the worldwide rate of dengue contaminations every year have extended between 50 million and 200 million; be that as it may, late gauges utilizing cartographic methodologies recommend this number is nearer to right around 400 million. Same relevance with another research revealed that Dengue has been present for centuries. The first recorded symptoms compatible with dengue were noted in a Chinese medical encyclopedia in 992 ADS, however originally pub¬lished by the Chin Dynasty centuries earlier (265–420 AD), prior to being formally edited 17, 18.

## Conclusion

In this research, it is concluded that the diagnosis of dengue cases is preliminary limited to initial stages i.e. CBC or sometimes dengue NS-I, as dengue IgM severity is more effective than that of Dengue NS-I & IgG, that why all parameters should be used for accurate diagnostic purpose of Dengue fever disease. It is humbly requested to physicians and doctors dealing with dengue outbreak every year should advance their diagnostic measures and should also pursue their prescription on complete and authenticated diagnosis of dengue patients.

## Abbreviations

CBC: Complete blood count; IgM: Immunoglobulin M; IgG: Immunoglobulin G; DENV: Dengue Virus; W.H.O: World Health Organization; DHF: Dengue hemorrhagic fever;
